# Comparison of Metrics for Investigating Bioequivalence in Onset of Action in Incurred Bioequivalence Studies

**DOI:** 10.3390/pharmaceutics18080988

**Published:** 2026-08-10

**Authors:** Paulo Paixao, Esperanza González-Rojano, Dolores Ochoa, Francisco Abad-Santos, Manuel Roman, Víctor Mangas-Sanjuan, John Gordon, Alfredo García-Arieta

**Affiliations:** 1 Research Institute for Medicines (iMed.ULisboa), Faculty of Pharmacy, Universidade de Lisboa, 1649-003 Lisbon, Portugal; ppaixao@ff.ulisboa.pt; 2 Jefatura de Docencia e Investigacion, Hospital Central de la Defensa Gomez Ulla, 28047 Madrid, Spain; 3 University Center for Health Sciences—HM Hospitals (CUHMED), Camilo Jose Cela University, 28692 Madrid, Spain; 4 HM Hospitals Health Research Institute, 28015 Madrid, Spain; 5 Clinical Pharmacology Department, Hospital Universitario de La Princesa, Instituto de Investigación Sanitaria la Princesa (IIS-IP), 28006 Madrid, Spain; 6 Pharmacology Department, Facultad de Medicina, Universidad Autónoma de Madrid, 28029 Madrid, Spain; 7 Department of Pharmacy and Pharmaceutical Technology and Parasitology, University of Valencia, 46100 Valencia, Spain; 8 Division of Biopharmaceutics Evaluation, Bureau of Pharmaceutical Sciences, Therapeutic Products Directorate, Health Canada, Ottawa, ON K1A 0K9, Canada; 9 Division of Pharmacology and Clinical Evaluation, Department of Human Use Medicines, Spanish Agency for Medicines and Health Care Products, 28022 Madrid, Spain

**Keywords:** bioequivalence, onset of action, time of maximum concentration, partial area under the curve until the median t_max_ of the reference, partial area under the curve until the t_max_ of the reference in each subject, absorption constant, intercept

## Abstract

**Background:** In those products where onset of action is clinically relevant, different metrics have been proposed to demonstrate bioequivalence, and different metrics, statistical methods and acceptance criteria have been employed in the United States, the European Union and Canada in the past. Recently, a harmonised approach has been recommended by the ICH M13A guideline. **Methods**: This work investigates the outcomes of several metrics in studies conducted in the Clinical Trials Unit of Hospital Universitario de La Princesa, Madrid, Spain, from 2000 to 2016, to identify the most adequate metric to reflect the onset of action. **Results**: An optimal single metric cannot be identified in these studies. The metrics show very high variability in most cases, making the conclusion of bioequivalence very unlikely with the usual sample sizes required for demonstration of bioequivalence based on Cmax and AUC. This is particularly evident if 90% confidence intervals are required to aim at inference of the study results to the whole population. Optimisation of the sampling times around tmax may be needed to reduce variability and increase reliability of the estimations. **Conclusions**: pAUC and tmax have been considered acceptable metrics under the ICH M13A guideline. For tmax, a non-parametric 90% confidence interval of the difference between the two formulations within a widened acceptance range may be sufficient for concluding BE in the early onset of action. Regarding pAUC, its high intra-subject variability can be addressed with the current scaling.

## 1. Introduction

The objective of bioequivalence (BE) is to assure that the drug levels and the shape of the drug concentration–time curve from the test product are sufficiently similar to that of the reference product. Drug levels are compared with metrics of systemic exposure. “Total exposure” or the extent of systemic exposure is compared with the area under the curve (AUC) of the systemic concentration–time profile and “peak exposure” with the maximum concentration of the systemic concentration–time profile (Cmax) [1,2,3]. These two metrics are enough to assure equivalent efficacy and safety for most drug products, but for those products where rapid or slow onset of action is clinically relevant, an additional metric addressing the shape of the ascending part of the curve is necessary. On the contrary, the shape of the descending part of the curve does not need to be compared in the case of immediate-release (IR) products because, except in the very rare case of flip-flop kinetics, that shape depends on the drug elimination half-life, and it does not depend on the drug product.

In the United States, the Draft Guidance for Industry “Bioequivalence Studies with Pharmacokinetic Endpoints for Drugs Submitted Under an ANDA” [4] recommended the use of partial AUC as an exposure measure if specified in the applicable product-specific guidance (PSG), and some modified-release products with different phases of release were mentioned as an example. In the Draft Guidance “Bioavailability Studies Submitted in NDAs or INDs—General Considerations” [5], partial AUCs are required, in addition to peak and total exposure, for certain classes of drugs (e.g., analgesic drug products). The time to truncate the partial AUC should be related to a clinically relevant response measure, and sponsors should consult the appropriate review division regarding the suitability of the pharmacodynamic (PD) measure or the use of partial exposure in general. Therefore, guidance regarding the truncation time is not available, unless specified in a PSG.

As of August 2020, the US FDA had issued 44 PSGs [6], but only one of them was an IR product for oral administration with systemic effect: Oxycodone HCl IR tablets. Early pAUC (0–3 h and 0–4 h) was required in the in vivo abuse deterrence study as supportive data only. As supportive evidence, there is no specific recommendation that the 90% CIs of the geometric mean T/R ratios for the early pAUCs should fall within the limits of 80.00–125.00%, but an upper limit greater than 125.00% may be concerning. The other IR product for which a pAUC was recommended as supportive evidence was a nasal spray of naloxone HCl (0–4 min, 0–10 min and 10–30 min).

Between August 2020 and August 2024, 18 additional PSGs have included pAUC [7]. Again, most of these products are extended-release products and a locally acting gastrointestinal product. Early pAUC for quick onset of action is also required for inhaled loxepine powder and naloxone HCl or nalmefene HCl nasal sprays.

This illustrates that pAUC is used mostly by the US FDA to assess the shape of the curve of extended-release products or the site of release of locally acting products in the gastrointestinal tract, but currently, the US FDA is not using early pAUC for the demonstration of BE of products containing drugs with a clinically relevant onset of action after oral administration (e.g., analgesics), except as supportive in the abuse deterrence studies of opioids.

The US FDA has acknowledged that for many drug products, data for characterizing the PK/PD relationship may not always be available to the FDA, and the identification of clinically relevant pAUC(s) in those cases could be challenging [6]. However, the time to truncate the partial area that is related to a clinically relevant measure is not a problem for pharmaceutical companies submitting in the USA, because it has to be stated in the PSG. In contrast, in other jurisdictions without PSG for all drugs, it may become a matter of controversy between applicants and regulatory assessors.

In Canada, the guidance document “Conduct and Analysis of Comparative Bioavailability Studies” [8] indicated that, when the time to onset of action is important, the area under the curve up to the tmax of the reference product, calculated for each study subject (AUCRef tmax), should be reported. The guidance document “Comparative Bioavailability Standards: Formulations Used for Systemic Effects” [9] stated that the relative mean area under the curve up to the time of the maximum concentration of the reference product (AUCRef tmax) for the test-to-reference comparison should be within 80.0% to 125.0% inclusive. Therefore, it is not assessed based on 90% confidence intervals. The AUCRef tmax ratio for each subject should be calculated using the test and reference values obtained for that same subject and not using a central value (mean or median) for the reference product.

In contrast, in the European Union, partial AUCs were not required for IR products. The “Guideline on the Investigation of Bioequivalence” [10] stated that a statistical evaluation of tmax is not required. However, if rapid release is claimed to be clinically relevant and of importance for onset of action or is related to adverse events, there should be no apparent difference in median tmax, and the variability (range) between test and reference product should also be similar. As tmax is extremely sensitive to the time of sampling as well as to the rate of drug absorption, the confidence intervals for this metric are generally too wide to be useful in determining BE [11]. In addition, tmax is not well defined in the case of multiple peaks or when the peak is flat [12].

Recently, the International Council for Harmonisation (ICH) published the M13A guideline harmonizing the scientific and technical aspects of study design and data analysis to support BE assessment based on PK endpoints for orally administered IR solid oral dosage forms [13]. The topic of early exposure was only marginally addressed, considering that both partial AUCs and tmax may be used for this purpose, but without providing any relevant direction on how to evaluate these metrics for the BE decision. As such, this topic still needs to be harmonised, probably in M13C, when addressing data analysis and BE assessment for more complex drugs [14]. Therefore, the purpose of this work is to compare the results obtained with these two and other alternative metrics for the comparison of the shape of the ascending part of the concentration–time curve, i.e., early exposure or absorption rate, to ensure equivalence in the onset of action.

## 2. Materials and Methods

### 2.1. Data Collection

The data presented in this report have been collected from the Clinical Trials Unit of Hospital Universitario de La Princesa (UECHUP), Madrid, Spain, from 2000 to the end of 2016. We have reviewed all clinical trials performed in this unit to select those IR products where the onset of action may be considered clinically relevant and where BE was investigated in a 2-sequence 2-period cross-over design. Other more complex designs were excluded due to the difficulty in clearly identifying the reference product tmax, e.g., in replicate designs where the two profiles with the reference product may exhibit a different tmax value. With these criteria, a total of 18 BE studies for 8 different drugs were selected for a total of 20 drug comparisons, because two fixed-dose combinations were investigated (ibuprofen/paracetamol and ibuprofen/tramadol). These 20 drug comparisons included analgesics (dexketoprofen (2), etoricoxib (2), ibuprofen (7), metamizole (1), paracetamol (1), and tramadol (1)), since analgesics are an example mentioned in the US FDA guidance [5], and phosphodiesterase-5-inhibitors (tadalafil (5) and vardenafil (1)), since the onset of action is considered relevant in the EMA product-specific guideline for tadalafil [15,16].

### 2.2. Study Design

All studies involved healthy male and/or female volunteers. The number of subjects included ranged from 18 to 48 (see Table 1). All studies were single-dose 2 × 2 designs. Before carrying out the trials, the protocol and informed consent form were reviewed by the Independent Ethics Committee in Clinical Research of the Hospital Universitario de La Princesa and the Spanish Regulatory Authority (Agencia Española de Medicamentos y Productos Sanitarios (AEMPS)). All documents were in accordance with current Spanish regulations, the International Conference on Harmonisation guidelines for Good Clinical Practice, and the Revised Declaration of Helsinki [17]. As usual, subjects were housed from 10 h before dosing with an acceptable washout period and received the test or reference formulation orally with generally 240 mL of water. Blood samples were collected, and plasma concentrations were analysed. The pharmacokinetic data used for BE were the classical primary metrics, maximum concentration (Cmax) and area under the curve from time zero to the last quantifiable concentration (AUC0-t), as well as partial AUCs truncated at different times around population median tmax of the reference product (pAUCtmax) and at the tmax of the reference product within each individual subject (pAUCRef tmax), as requested in Canada [8,9]. Different truncation times around the median tmax of the reference product were investigated for information and because a sampling time with pharmacodynamic relevance might not be agreed upon, and we have not identified any in the US FDA product-specific guidance for these drugs [18]. In principle, partial AUCs until one sampling time before the population median tmax of the reference, the population median tmax of the reference (pAUCtmax, which is considered similar to the US FDA approach [3] in the absence of a time point with PD relevance) and few sampling times after that population median tmax of the reference were estimated. In addition, tmax was analysed “visually” based on the similarity of the median and range, as recommended by the EMA [10] in the past, based on non-parametric 90% confidence intervals assessed as differences [19,20]. Since a non-parametric test does not allow assessment of the variability in the data, the parametric 90% CIs for the log-transformed tmax values were also calculated in order to have an idea of the within-subject variability in that metric, for information purposes only. In addition, the metrics Cmax/AUC0-t [21,22,23,24], the absorption rate constant (ka) [25], the mean residence time (MRT) [26] and the intercept [27,28,29] were calculated to obtain a whole picture regarding the rate of absorption and to support the decision on the most adequate metric to compare the rate of absorption, shape of the concentration time curve and onset of action between test and reference products in each of these BE studies. The absorption rate constant for each individual profile was determined based on the observed tmax and elimination rate constant (ke) and by minimizing the difference between the numerical solution of the equation tmax = ln(ka/ke)/(ka − ke) and the observed tmax, using the solver from Microsoft Excel. MRT was calculated based on the equation MRT = AUMC/AUC. The intercept was calculated according to Csizmadia and Endrenyi [30] by determining the linear intercept of the ln(C/(t-tlag)) vs. (t-tlag) plot. In this situation, the tlag was estimated by fitting a line to the first two plasma concentrations that are above the limit of quantitation and taking the intersection of the line with the time axis.

### 2.3. Statistical Analysis

Mean plasma concentration profiles were compared in each study, based on a visual assessment, and organised in 3 classes (“similar”, “slightly different” and “different”).

The pharmacokinetic metrics were estimated by noncompartmental analysis with the linear trapezoidal rule and the nominal sampling times since the sampling time deviations were negligible for our purpose.

The parametric 90% confidence intervals for the ratio test/reference of these pharmacokinetic metrics were calculated after log-transformation. These calculations considered the following factors in the model: sequence, subject (sequence), period, and formulation [31]. All calculations were conducted in Microsoft Excel for Mac (version 16.16.27).

The non-parametric 90% confidence intervals of the difference between the test and reference tmax were calculated as described in the literature [19,20].

For interpretation of the different metric relationships using the totality of data, a log-transformation of all the metrics followed by within-study normalisation by the formula (X-average)/standard deviation was performed. The data from all the studies was then merged, and a correlation analysis performed.

## 3. Results

**Figure 1:**
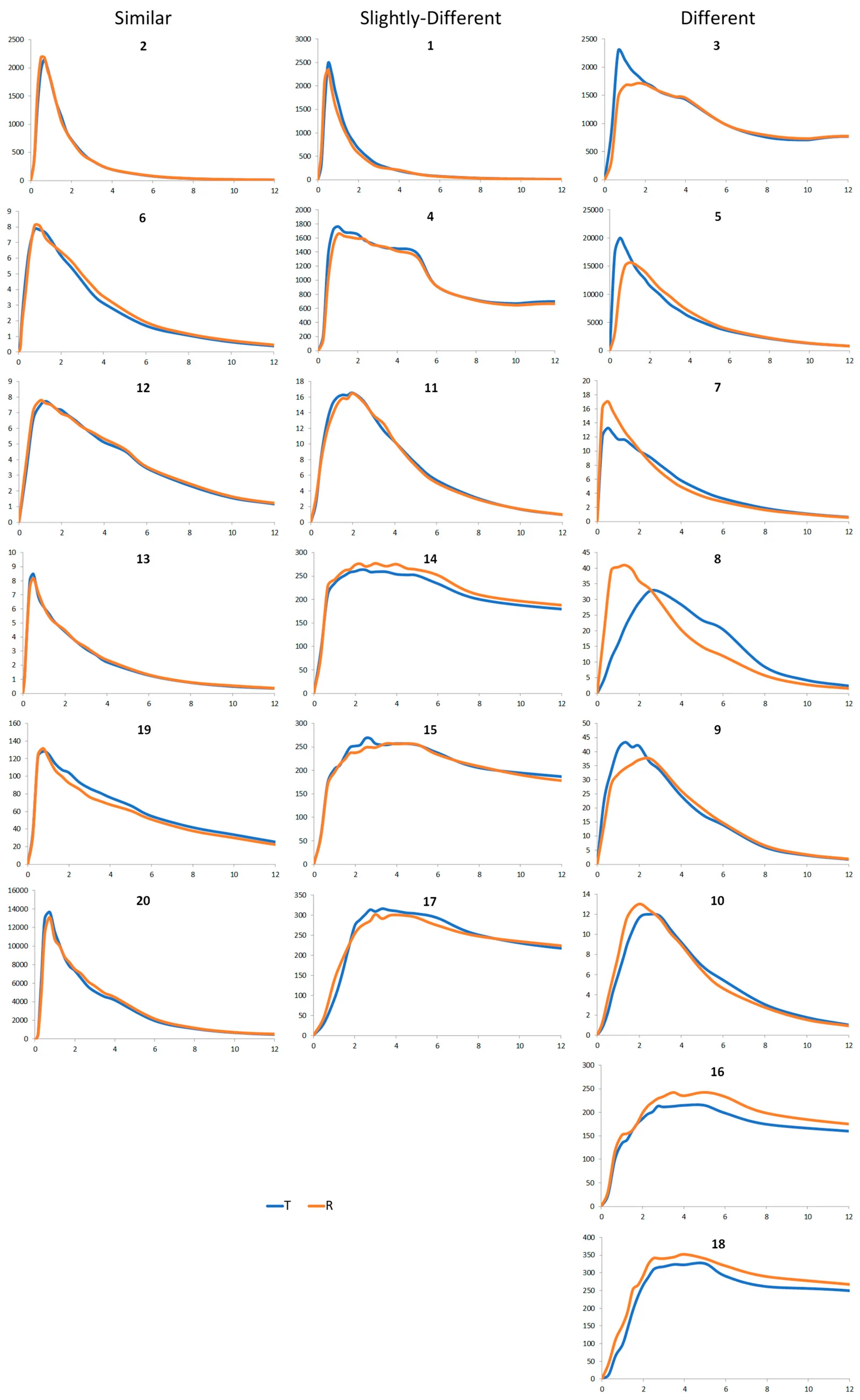
Mean profiles between the test and reference products in each study, up to 12 h post-administration.

Table 1 shows the results of the 2 × 2 cross-over BE studies conducted between 2010 and 2016 in Hospital Universitario de La Princesa, where the onset of action was considered clinically relevant. These cases, as organised by the visual assessment of the similarity of the shape of the mean profiles between the test and the reference products in each study, are also presented in Figure 1.

In four studies, non-BE was concluded by the usual Cmax and AUC metrics (#3, #7, #8 and #16), even without taking into consideration the early onset of action. These cases were included in the group of profiles considered “different” by visual inspection in Table 1. Four other cases (#5, #9, #10 and #18) were also included in this group because they exhibited profiles that visually differ around the absorption phase, although Cmax and AUC were able to show equivalence. It is considered that these eight cases represent performance differences that are probably relevant.

In the “slightly different” group, six cases were included (#1, #4, #11, #14, #15, and #17), because the profiles are not superimposable in the absorption phase. In this case, it was considered that these products might have similar performance. Finally, six cases (#2, #6, #12, #13, #19 and #20) were included in the “similar” group since the profiles were superimposable in the absorption phase and the performance was considered to be the same.

Taking into consideration the MRT and Cmax/AUC as alternative metrics to assess the “early-onset” of action, these metrics had less variability, but also less sensitivity to detect differences between formulations. Non-BE was only concluded in one case by using MRT (#8, ibuprofen 4) and in two cases by considering the Cmax/AUC metric (#7 and #8, ibuprofen 3 and 4). These cases have already been concluded as non-BE by the Cmax and AUC general criteria.

Regarding tmax, just looking at differences in the test and reference median and range values, besides its subjective nature, does not seem to be able to increase discrimination ability either. In only two cases (#5 and #8, ibuprofen 1 and ibuprofen 4), it was possible to unequivocally observe notable (more than two-fold) differences, and in one of these, BE has already been rejected based on the Cmax.

If the 20% acceptance criterion is applied to tmax, as defined in the product-specific guidelines of ibuprofen [32], paracetamol [33] and tadalafil [15], tmax becomes more discriminatory, perhaps over-discriminatory. Two more cases are detected within the “different” group (#3 and #7, etoricoxib 1 and ibuprofen 3), with the former exhibiting a 33% difference (20 min), but this refers to adjacent sampling times. The latter had failed the acceptance range for Cmax, and the 33% difference (7.5 min) refers to less than two adjacent sampling times (15 min) since the median of the reference tmax is in between two sampling times. In these two cases, the difference in shape that is detected visually is actually caused by the different peak level, but the ascending slope is not so different visually (see Figure 1). One case is detected in the “similar” group (#6, Ibuprofen 2), where the difference (15 min) at adjacent sampling times was 33% of the tmax of the reference, which might be explained because the sampling times were not frequent enough. This illustrates that when tmax is expected so early, samples should be taken more frequently (e.g., every 5 min). Finally, two cases are also identified in the “slightly different” group (#14 and #15, tadalafil 1 and 2). In the first case, the difference (21%, 22.5 min) is larger than two adjacent sampling times (15 min), which indicates a real, although small, difference. In the second case, the difference (43%, 45 min) is notably larger than between adjacent sampling times (15 min).

All the remaining metrics and statistical tests were much more discriminatory, by concluding non-BE in multiple cases, but also subjected to much more uncertainty. In all situations, the estimation of the within-subject variability was always higher than the one observed for Cmax.

Considering the cases grouped as “similar”, it can be observed that the tmax evaluated by the non-parametric 90% CI for the difference between the test and reference are contained, except in one case, inside an acceptable difference of 20 min and also one third of the median reference tmax (i.e., 13.4, 15*, 20, 10, 15 and 15 min, respectively). This exceptional case (#6) is the same as what was detected by the comparison of the medians with a 20% acceptance range, and it seems to be caused by infrequent sampling, which seems to be responsible for one of the largest intra-subject CV(%) values (45%), based on the parametric 90% CI. It should be also noted that the median reference tmax values for all cases in this group were less than or equal to 1 h. Still regarding tmax variability in the “similar” group, a medium-to-high value was observed (CV of 30% to 46%). These values were generally lower than those observed in the remaining metrics of early exposure. Of these, the intercept metric provides similar conclusions to the non-parametric 90% CI for tmax differences, although, in most cases, with even higher variability (CV of 18% to 63%). Regarding ka and the two pAUCs, their parametric 90% CI for the T/R ratio mostly includes the 100% value, although they do not conclude BE due to (very) high variability in most cases (CV of 36% to 79% for ka, 24% to 90% for pAUCtmax and 24% to 65% for pAUCRef_tmax).

From the cases considered visually “different”, it can be seen that all the metrics conclude non-BE except in one case, where the non-parametric tmax concluded BE within plus/minus one third of the median reference tmax (#16, tadalafil 3), i.e., 60 min, but in this case, non-BE has already been concluded by the Cmax (a mandatory metric). In fact, in that case, the difference in the shape of the curve is more related to the peak exposure than to the ascending slope, like in other cases of this group. In general, the within-subject variability was high in all metrics but, again, usually lower for the tmax parametric 90% CI.

In the “slightly different” profiles, good agreement between all metrics can be observed, and BE was not concluded, mostly due to high variability, as the 90% CI usually includes the 100% or 0 min difference. The exception is the pAUCRef tmax, where a clear non-BE was concluded in two cases (#1, dexketoprofen 1 and #17, tadalafil 4). This can be an indication of higher sensitivity but at the cost, again, of very high variability. Of relevance, the calculations for the 90% CI of pAUC were not possible to be performed in two cases (#1, dexketoprofen 1 for pAUCTmax and #4, etoricoxib 2 for pAUCRef tmax).

**Figure 2:**
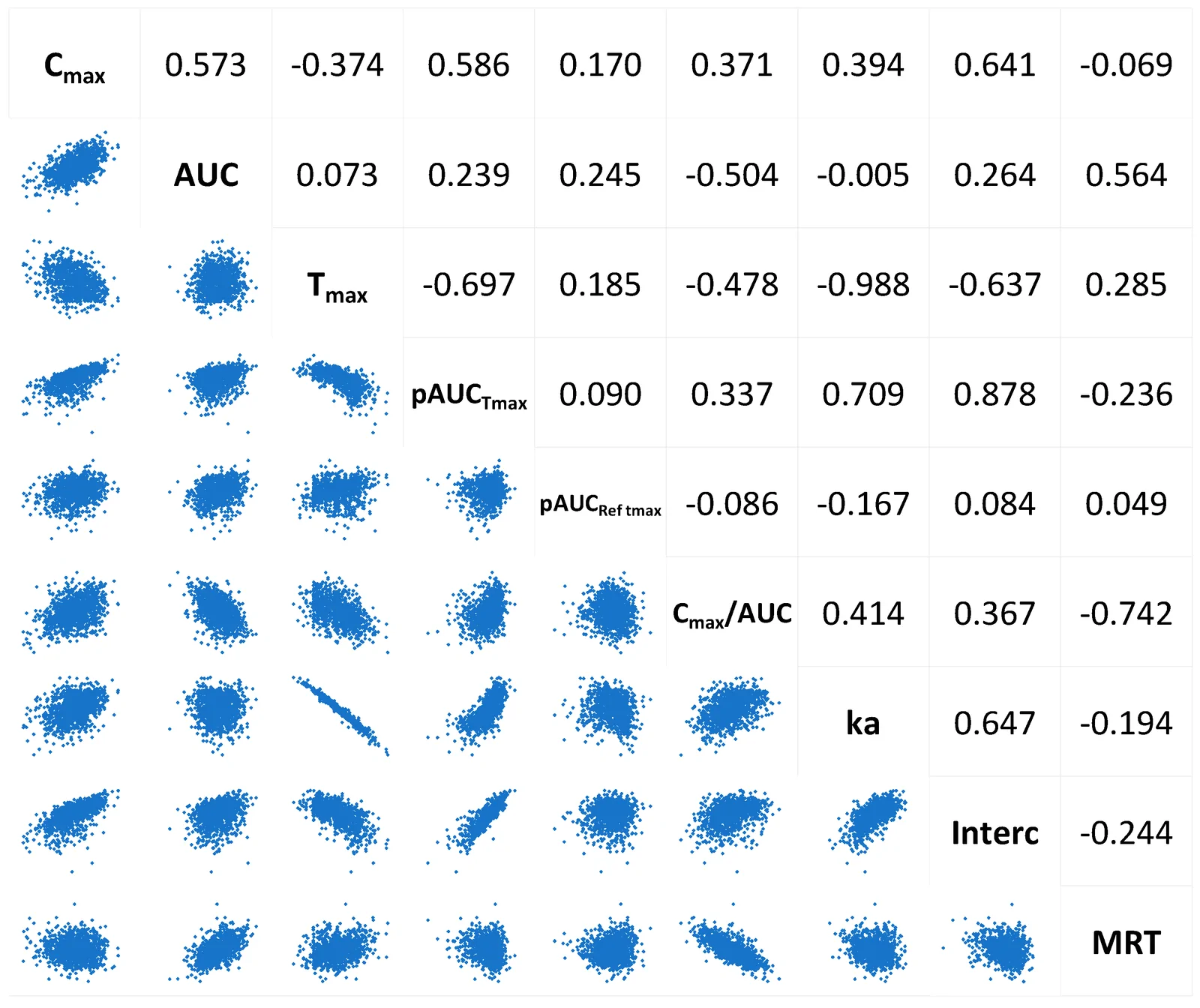
Correlation analysis between all the PK parameters normalised within study.

The comparison of the different metrics from all the studies after log-transformation and within-study normalisation is presented in Figure 2. It can be seen that Cmax is correlated with AUC and tmax, but AUC and tmax are not correlated. This agrees with the PK theory, where tmax is considered a rate metric, AUC is an exposure metric and Cmax is both a rate and an exposure metric. The pAUCtmax is also strongly correlated (positively) with Cmax and (negatively) tmax. In fact, although being an exposure metric, this partial AUC seems to describe the same information as the tmax metric. The same can be concluded for ka being very strongly correlated with tmax and intercept metrics being strongly correlated with pAUCtmax. The Cmax/AUC and MRT metrics were slightly more correlated with AUC than with tmax. Finally, the pAUCRef tmax, on the contrary, is not strongly correlated to any of the other PK metrics. This is somewhat unexpected, as it should generally characterise the same type of information as the pAUCtmax, especially under the presented examples, which are all IR oral products with simple kinetic processes.

Finally, the impact of calculating partial AUCs truncated at different time points around the population median tmax of the reference product (pAUCtmax) is presented in Table S1. The T/R ratios are usually farther from unity when earlier truncation times are used, but this deviation decreases progressively as the truncation point moves to times later than tmax. A similar trend is observed in the variability of the comparison: variability is markedly higher with early truncation points and decreases substantially as later points are used. Consequently, later truncation yields more stable estimates but reduces sensitivity to detect differences between formulations, leading to conclusions that increasingly resemble those obtained with the full AUC0-t.

## 4. Discussion

The review of the BE studies conducted in Hospital de La Princesa between 2010 and 2016 with products in which the onset of action could be considered clinically relevant shows that the different methods employed in the USA, the EU and Canada do not behave consistently. This difference could be expected when contrasting the EU’s “visual” assessment of tmax with the pAUC-based approaches used in the USA and Canada. However, a similar behaviour would also be anticipated when comparing partial AUCs truncated at a common time point for all subjects (e.g., the median tmax of the reference) with those truncated at the individual reference product tmax, as required in Canada. Notably, several examples demonstrated markedly different test/reference point estimates between these two methods (e.g., ibuprofen 2, 5, 6 and 7; paracetamol; tadalafil 1, 2, 4 and 5; and vardenafil). This different behaviour is also observed in the correlation analysis (Figure 2), where the two partial AUCs are weakly correlated with each other.

Importantly, in 1986, Concheiro et al. concluded that if the aim is to detect differences in the rate of absorption, even though these differences may not affect the overall plasma profiles, the partial AUC (pAUC) is the adequate metric [34]. This conclusion was confirmed by Romero et al. in 1990 [35], who concluded that several truncated AUC ratios could provide meaningful information on absorption rates for BE testing. In some of the cases considered “different” in the present investigation, the pAUC tmax seems to be the most adequate (etoricoxib 1), but in other examples, the non-parametric 90% CI of tmax based on differences seems to be more adequate (ibuprofen 6), because the high variability makes pAUCtmax uninformative. On the other side, pAUCtmax seems to be more discriminating than the non-parametric 90% CI of tmax differences in the only case where mean profiles were considered “similar” visually, but non-BE was concluded by the metrics (ibuprofen 2). In the “slightly different” group, the point estimate of pAUCtmax in one case (tadalafil 2) seems over-discriminative compared to the non-parametric 90% CI of tmax, but the high variability observed precludes any conclusion. In fact, there is a strong correlation between tmax and AUCtmax, and these two metrics are, basically, assessing the same profile characteristics (Figure 2). pAUCRef_tmax, in addition to not being correlated with the previous two metrics, is also shown to be over-discriminating in other cases (dexketoprofen 1 and tadalafil 4); however, this does not seem to be a correct decision based on the mean plots of Figure 1.

The pAUCs up to a fixed sampling time as applied in the United States, or up to tmax in the reference product for each subject, as applied in Canada, exhibit an important drawback: i.e., the study would not be assessable simply if the pAUC in one profile is zero and log-transformation is not possible. The exclusion of that subject would not be acceptable, since it would contribute to equivalence, and the imputation of a value would be arbitrary and unjustified, and would most likely cause the study to fail. This problem was observed in two cases with truncation at a fixed sampling time: once with truncation at the median tmax (1 out of 20, i.e., 5%) and once with truncation at the tmax of the reference in each subject (5%). This Canadian criterion has additional limitations since it cannot be used in case of parallel designs, and in case of replicate designs, the tmax values in both periods of the reference may differ.

The second limitation of pAUCs is that they are usually extremely highly variable, and this large variability would require a replicate design to scale based on the intra-subject variability observed in the reference product. The widening employed in the European Union, with a maximum widening of up to 50% CV, would not be enough to maintain the sample size within reasonable numbers. Therefore, pAUCs would require the elimination of that maximum widening. The replicate design could be avoided if the acceptance ranges were widened, as proposed by Rostami et al. [1], to a fixed acceptance range (e.g., 75.00–133.33%) instead of widening it based on the intra-subject variability of the reference product. However, a 25% acceptance range would not be enough for the extremely high variabilities observed in these studies, and much larger sample sizes would be required even with those widened fixed limits.

An alternative approach to deal with high variability could be the one taken in the previous Canadian criteria, where the point estimate of the ratio test/reference is used instead of the 90% confidence intervals. However, this is no longer a possibility for Cmax, according to the ICH harmonisation [13], as it lacks the methodological or statistical requirements to infer from the sample of a small BE study to the whole population of potential patients.

The definition of the pAUC, as it has been used in the United States and as stated in the ICH M13A guideline, is problematic for pharmaceutical companies because it is not possible to know in advance the truncation time for the pAUC desired by the regulatory agency, unless it is predefined in a PSG. In the US, this approach is applicable because the pAUC is only recommended in those cases identified in the PSG, but in the rest of the jurisdictions, where no PSG or few PSGs are available, it cannot be assumed that pAUC is not necessary. In these jurisdictions, it would be much more pragmatic to truncate at the median tmax of the reference product instead of based on the pharmacodynamically relevant sampling time, which might be controversial. However, such an approach is not applicable in many cases, because the time of onset of action and tmax are notably different (e.g., carbamazepine [36]). The rate of absorption of carbamazepine is clinically relevant due to the incidence of adverse events in the first two hours after administration if the rate of absorption is too rapid, but tmax occurs much later (11 h), and it might be unrelated to the shape of the curve that affects the onset of action. Even in the case of tadalafil, the observed tmax in the fasting state occurs much later than the onset of action (1.75 to 3 h vs. 16 to 30 min) [37].

The European Union criterion, based on the “visual” assessment of tmax medians and ranges, lacks objectivity and is inherently open to interpretation. Even though some objectivity was recently introduced by requiring the T/R ratio of the medians to fall within the 80–125% acceptance interval [15], this approach still lacks robust methodological and statistical foundations. More importantly, several cases in the “different” group were identified in which neither the tmax medians nor their ranges were able to indicate any meaningful difference (ibuprofen 5 and 6 and tadalafil 3 and 5).

However, when a non-parametric 90% CI is applied, the subjective component is removed, and its ability to discriminate between formulations becomes comparable to that of the pAUC metrics. Indeed, there is a clear relationship between the conclusions drawn from the non-parametric 90% CI for tmax and the visual assessment of similarity between mean plasma concentration–time profiles. When profiles are “similar” or only “slightly different,” these 90% CIs generally include zero. By contrast, in profiles classified as “different,” the 90% CIs frequently exclude zero or zero is just at the boundary of the 90% CI, and therefore, they clearly indicate non-BE.

The main concern associated with the use of non-parametric 90% CI for tmax is the arbitrary definition of an acceptance range. Ideally, it should be defined individually in product-specific guidelines based on the available PK/PD data for each specific drug. However, this may not be feasible, and therefore, an arbitrary acceptance range should be proposed by default, which might be modified by the corresponding PSG in specific cases. It seems feasible to maintain an acceptance range of one third of the median reference tmax, i.e., 33.3%, except in those cases where tmax is observed sooner than 1 h, where 20 min could be used as the narrowest acceptance range for tmax. Therefore, the narrowest acceptance range would be 20 min when the median tmax of the reference product is 60 min or less, but it widens gradually from that time onward. This 20 min acceptance range is a compromise between feasibility and clinical relevance. The tmax differences between ibuprofen lysinate and ibuprofen acid can be as short as 20 min (33 min vs. 53 min) [38], and this difference is reflected in the onset of the analgesic effect, which was considered clinically significant in one study [39], although not detected in another study [40]. It is expected that tmax differences of 20 min will not be reflected in differences larger than 5 min in onset of action, which can be considered the minimum difference with clinical relevance [41,42,43]. Differences with clinical relevance have been observed when tmax differences were larger than 20 min. A difference in tmax of 40 min (24 vs. 63 min) between the ibuprofen arginine granulate and ibuprofen acid tablets at a strength of 400 mg produced an analgesic effect significantly quicker and higher than that of ibuprofen tablets [44]. Also, in a comparative study between a glycinated aspirin and soluble aspirin in migraine, patients resulted in a tmax difference of 25 min (1.14 h vs. 0.73 h), which showed numerical superiority for the soluble aspirin with faster tmax and higher Cmax, but there was no significant difference between the two treatments with respect to pain relief due to the small sample size of 10 migraine patients [45]. As such, it seems that a tmax difference of 20 min could be considered an acceptable cut-off point for a regulatory acceptance criterion. However, in all these previous cases, the reference tmax was lower or around 1 h. In a comparative study between two different naproxen formulations where the fastest (naproxen sodium) presented a tmax of 1 h and the slowest (naproxen) a tmax of 2 h, pain intensity was consistently lower for the group receiving naproxen sodium. However, statistically significant differences were not seen until 4 or 5 h after medication due to the limited sample size of 30 postpartum women [46]. Therefore, an acceptance range of one third of the median reference tmax, i.e., 33%, for studies where the reference product exhibits a median tmax equal to or larger than 60 min, which allows for increased acceptance limits, e.g., 5 min increments over time from that time onward.

Applying the proposed acceptance limits of 33% to the non-parametric 90% CI of tmax shows that all but one of the studies classified as “similar” (ibuprofen 2) fall within the proposed bounds. This group includes dexketoprofen 2, metamizole, paracetamol, tramadol, and vardenafil, with reference median tmax values between 0.5 and 1 h. Among “slightly different” products, 50% met the proposed criteria for BE (dexketoprofen 1 and tadalafil 2 and 4), while the remaining cases failed only marginally (24.9 vs. 23.4 min for etoricoxib 2, 37.5 vs. 35 min for ibuprofen 7 and 37.5 vs. 35 min for tadalafil 1), most likely—because the intervals still included zero—reflecting poor tmax characterisation, since samples were not sufficiently frequent, and high residual variability rather than genuine formulation differences. This indicates that the proposed limits are not overly permissive or conservative, and that some studies may still require optimisation in sampling times or sample size.

Finally, for products classified as “different,” all except one did not meet the proposed tmax criterion, as expected. The remaining case (tadalafil 3), although meeting the tmax limits, failed on Cmax, and it was thus not bioequivalent. This group of “different” profiles included drugs with reference median tmax ranging from 0.375 to 3.5 h.

Regarding the remaining metrics, in particular MRT, the results of this study agree with the conclusions by Khoo et al., who reported in 1985 that, in some situations, this metric was less sensitive than Cmax and tmax in detecting differences in rates of absorption [47]. Its limited usefulness was confirmed by Aarons in 1987 [48]. The time profile of the absorption rate is insufficiently characterised by only one metric, such as the mean absorption time (MAT) [28]. Therefore, MRT can be discarded as an alternative metric for assessing equivalence in rate of absorption and onset of action. Cmax/AUC was recommended in 1991 by Endrenyi et al. [21] for assessing the equivalence of the absorption rate because it reflects only the contrast between the absorption and disposition rate constants and is independent of the extent of absorption. In addition, it is less variable than Cmax, and its variability does not depend on the variability of the extent of absorption [22]. Therefore, preferable to Cmax [23]. However, the authors acknowledged that the ratio Cmax/AUC shares with Cmax its insensitivity in representing changes in the absorption rate constant and suggested the need for other measures, like pAUCs, for this purpose [21]. This was confirmed by Bois et al. [49], who noticed that when bioavailability is low, Cmax may be better than Cmax/AUC0-inf and is less sensitive to changes in rates than pAUC. Tozer et al. [2] also discouraged the use of Cmax/AUC because it is not sensitive to changes in rate of absorption and BE can be declared more easily because its variability is lower even when Cmax is not equivalent, which raises safety concerns. This is observed in some cases in this study (e.g., etoricoxib 1 and 2 and ibuprofen 1), where we have used Cmax/AUC0-t, as it is preferable to Cmax/AUC0-inf because the latter is sensitive to measurement errors [24]. However, Endrenyi and Tothfalusi proposed Cmax/AUC0-t as an alternative to Cmax [21,50], not as an additional metric to assess rate of absorption or the shape of the ascending part of the concentration–time curve. However, the need for Cmax as peak exposure and as a safety measure is undeniable.

Endrenyi and Al-Shaikh also identified the intercept metric as a metric with very favourable kinetic and statistical properties to assess the equivalence of absorption rates in linear kinetic systems assuming mono-exponential disposition with first-order absorption [27], and Ring et al. assumed an inverse Gaussian model [28]. This intercept metric was improved further by Macheras et al. [29], but at that time, the focus had been changed from rate of absorption to “early exposure” [1,2]. These two intercept procedures showed higher kinetic sensitivity, but also higher variability than pAUC [51]. This study confirms that the intercept usually exhibits large variability, but the same problem can be observed in the case of pAUCs. That is also the behaviour observed for the ka metric, which also has the limitation that its determination assumes mono-exponential disposition with first-order absorption and, thus, may not be appropriate for all drug products.

In 1994, Bois et al. [49] compared the reliability of multiple metrics and concluded that there is no universal metric of rate. All rate measures fail in one aspect or another. In fact, none of them were able to detect a 25% difference in the absorption rate constant, and although tmax had good sensitivity, it also yields very high producer risks. In our data, the non-parametric 90% CI of tmax is a good metric in many cases, as explained above, but a higher sample size seems to be necessary to show equivalence, because its variability is much larger than the usual pharmacokinetic metrics for extent of absorption (AUC) and peak exposure (Cmax). These authors also concluded that Cmax and Cmax/AUC0-inf have low sensitivity, as we also see in many of our case examples. Regarding pAUCref_tmax, the same authors concluded that it has high sensitivity and potentially the best behaviour of all measurements, except in the case of random lag times. In our data, and as stated above, this metric seemed to be over-discriminating, and its variability is not consistently lower than that observed for tmax.

Due to the same lack of a clear path to take, Dokoumetzidis et al. [52] discouraged the use of a global, unique metric for assessing early drug exposure due to the complexity, nonlinearity and diversity of the pharmacodynamic phenomena. In contrast, they advised considering each drug individually and basing the appropriateness of the utilised metric on a large number of PK-PD data and well-established relationships between the proposed early exposure metric and the onset and/or intensity of action, as the FDA has proposed. In this line of thought, other investigations explored in more detail pAUC ratios as indicators of absorption rates and their relation to the cut-off point [12,49]. In 1992, Chen investigated various cut-off points around tmax for a drug since the absorption process takes place within a short time span and is nearly complete when the plasma level reaches its peak in most IR products [12]. Some common time points before and after tmax were calculated in addition to, e.g., pAUCRef tmax. In the case of two cephradine products with apparently similar absorption rates based on the shape of the concentration–time profiles, the pAUCs were too variable to conclude equivalence. This same limitation is observed in most of our presented case examples. In another case with trazodone, the rate of absorption looked similar, and pAUCRef tmax was used to conclude BE due to low variability. In another example with ibuprofen, the profiles suggested a different rate of absorption, and Cmax was insensitive to these differences and indicated equivalence, and pAUCRef tmax was too variable to detect the difference. In our study, we have also observed that, in some examples, the variability of pAUCRef tmax was moderate or high, whereas in other examples, it was extremely high. Finally, tolbutamide was used as an example of a drug with slower absorption, and the case products differ in their rates of absorption, although Cmax was equivalent. pAUCRef tmax was able to detect the difference despite the large variability. In these case examples, the authors also observed that the farther the cut-off point from the peak time, the tighter the confidence interval becomes and the lower the sensitivity for detecting differences (i.e., the more centred the point estimate is located). This is also observed in most of our cases where pAUCs were calculated at different cut-off points, as seen in Table S1. This highlights two issues regarding the use of pharmacodynamic-based endpoints: (1) if this results in a time before tmax, larger variability is anticipated, creating more difficulties in concluding BE, and (2) if the time is later than tmax, lower variability is expected, as well as a reduced ability to see differences in the two rates of absorption. This was also confirmed by Endrenyi et al. [53] and Macheras et al. [54], leading to the overall proposal that the optimal cut-off point would be the tmax of the reference formulation.

One of the limitations of this investigation is that these BE studies were not designed for the calculation of these metrics, especially ka, intercept and early exposure pAUCs, since they were conducted to obtain a marketing authorisation in the EU based on Cmax, AUC and the subjective and insensitive “visual” inspection of tmax. Therefore, the number of sampling times before tmax is usually insufficient for an adequate estimation of these metrics, and therefore, samples were not taken frequently in the first hour after administration (e.g., 5 and 15 min after dosing, followed by additional samples (e.g., two to five) in the first hour after dosing) [4]. In one study (ibuprofen 3) only, the median reference product tmax occurred before the second sample, excluding baseline. In four studies, pAUCtmax was calculated with two samples before population reference tmax, in three studies with three samples, in four studies with four samples, three studies with five samples, two studies with six samples, and one study with 8, 10 and 11 samples before the population reference tmax. Surprisingly, in the study where the median reference tmax occurred between the first and second sample post-dose (15 and 30 min, ibuprofen 3), the intra-subject variability was not high for pAUCtmax, pAUCRef tmax and the three other pAUCs calculated up to 1 h post-dose. Apart from this case, an improved sampling schedule is expected to reduce intra-subject variability. This case with low variability in pAUCtmax shows that the point estimate of the non-parametric 90% CI of tmax is more discriminative than that of pAUCtmax (15 min difference with respect to a reference tmax of 22.5 min, i.e., a difference of 33.3% in tmax vs. 20.4% in pAUCtmax). In the other case, where residual or intra-subject variability of pAUCtmax was low (23.97%, metamizole), the difference in the point estimate of the non-parametric 90% CI of tmax was zero, with the median reference tmax at 1 h; the point estimate of pAUCtmax was 89.39%; and the 90% CI was almost bioequivalent (79.50–100.50%). It could be said that pAUCtmax was more discriminative in this case, but perhaps it was over-discriminative, and when variability was low and profiles were “visually similar” (metamizole), tmax was able to show equivalence in contrast to pAUCtmax, and when profiles were different (ibuprofen 3), tmax was able to detect it better. Therefore, tmax is more predictive, or at least not worse, than pAUCtmax in the two cases with the best-case scenario of low variability for pAUCtmax.

Other drugs could potentially have been considered in this investigation. However, given that the assessment of the equivalent rate of absorption is required for a very limited number of IR drug products (e.g., none in the US FDA PSG for fasting state study of IR oral products and only for triptans, inhibitors of PDE5 and some analgesics in other jurisdictions), the selected drug products were considered the least controversial examples. Nevertheless, we acknowledge that, in some jurisdictions, the equivalence in the rate of absorption is not requested for some of these drugs. This is despite the fact that the rate of absorption is known to be important where quick onset of pharmacological action is required (e.g., hypnotics, anaesthetics) and even where it is known that the drug (i.e., antianxiety) needs to be dosed for some time before obtaining the desired therapeutic effect [55]. In addition, the onset of action may also be clinically relevant for avoiding adverse events when a slow input rate is needed [52] (e.g., antihypertensive drugs or carbamazepine).

## 5. Conclusions

It is difficult to identify a metric to investigate the equivalence in onset of action in those products containing drugs where onset of action is clinically relevant. Some of these studies suggest that pAUCtmax may be a suitable metric, but in other cases, the non-parametric 90% CI of tmax differences may be more adequate. In fact, there is a strong correlation between tmax and pAUCtmax, meaning that the two may be used interchangeably. It seems that pAUC up to a time with clinical relevance could be a preferred metric, but its high variability, especially if the cut-off point is very early, might require a replicate study design to scale the results/acceptance range. The 90% non-parametric confidence interval of tmax differences could be used as an alternative, if tmax is not too distant from the time with clinical relevance, to avoid high variability and the need for a replicate design, or to have as a rescue strategy of the study if pAUC is not calculable due to the absence of plasma levels at the cut-off point in any profile. However, if tmax is far from the time with clinical relevance, it loses its predictive value and sensitivity (e.g., carbamazepine).

Both approaches are limited by the high variability observed. This implies that more frequent sampling is necessary to improve estimation and reduce variability, and larger sample sizes might be necessary since these metrics are more variable than the usual pharmacokinetic metrics employed to demonstrate BE. Imposing some less demanding criteria, like accepting differences up to ±33% of the median reference tmax, up to a minimum of 20 min, in the non-parametric 90% confidence interval for differences in tmax may be necessary.

## Figures and Tables

**Table 1:** Test/reference point estimate, parametric 90% confidence interval of the ratio test/reference and intra-subject variability (CV(%)), except for tmax where, in addition, median and range for test and reference are shown separately and non-parametric 90% confidence intervals of the test/reference are calculated based on test–reference differences.

No.	Drug (Sample Size)	AUC_t_	C_max_	C_max_/AUC	MRT	Reference t_max_ Median (Range)	Test t_max_ Median (Range)	t_max_ as Differences in Min	t_max_ (Parametric)	ka	Intercept	pAUC_Tmax_	pAUC_Ref tmax_	
2		99.61	101.26	101.36	99.54	0.67	0.67	3.75	105.96	91.04	98.16	85.01	81.32	Similar
	Dexketoprofen 2	96.31–103.02	89.81–114.18	90.98–112.91	95.12–104.17	(0.33–1.75)	(0.33–3.00)	−4.8–9.9	93.83–119.66	76.42–108.44	83.05–116.02	69.86–103.44	67.92–97.35	
	(n = 35)	8.33	30.33	27.19	11.28				30.77	45.39	43.18	51.48	46.75	
6		95.78	91.48	95.51	96.31	0.75	1	0	98.59	104.27	104.78	129.48	107.31	
	Ibuprofen 2	91.74–99.99	83.78–99.88	88.75–102.78	93.48–99.23	(0.33–2.50)	(0.33–2.00)	−30.0–15.0	79.65–122.03	73.96–147	87.58–125.37	101.79–164.70	91.60–125.71	
	(n = 24)	8.7	17.88	14.91	6.04				45.2	78.69	37.48	51.54	32.76	
12		95.8	98.33	102.64	95.7	1	1.125	0	107.85	87.91	96.14	89.39	86.22	
	Metamizole	86.35–106.29	94.39–102.44	94.66–111.29	85.79–106.76	(0.67–2.67)	(0.67–2.33)	−12.6–15.0	89.02–130.67	67.35–114.74	87.82–105.25	79.50–100.50	76.86–96.73	
	(n = 24)	21.18	8.27	16.47	22.37				40.29	57.99	18.45	23.97	23.51	
13		98.42	103.11	104.77	98.1	0.5	0.5	0	103.38	95.84	112.04	111.19	85.23	
	Paracetamol	94.01–103.03	91.27–116.49	94.65–115.98	94.42–101.92	(0.17–2.00)	(0.33–1.25)	−5.1–5.1	86.27–123.89	75.59–121.53	90.05–139.39	88.61–139.51	63.90–113.68	
	(n = 24)	9.27	24.99	20.76	7.74				37.83	50.89	46.39	48.29	63.36	
19		110.11	104.65	95.04	99.49	0.75	0.75	7.5	116.33	80.05	96.83	100.93	99.95	
	Tramadol	103.13–117.56	95.92–114.18	88.13–102.49	95.75–103.39	(0.50–1.67)	(0.50–2.00)	0–15.0	102.52–132.01	67.1–95.5	86.37–108.56	84.85–120.06	82.48–121.12	
	(n = 24)	13.26	17.71	15.34	7.77				25.97	36.83	23.42	36.11	40.26	
20		95.08	99.69	104.84	95.67	0.75	0.75	0	96.53	107.38	99.35	119.12	90.58	
	Vardenafil	88.61–102.02	89.21–111.40	96.48–113.93	92.62–98.81	(0.33–2.33)	(0.33–2.33)	−7.5–7.5	82.46–112.99	85.64–134.63	80.65–122.4	90.44–156.88	73.14–112.18	
	(n = 44)	19.82	31.69	23.51	9.05				46.16	69.93	63.49	89.53	65.28	
1		106.83	99.08	92.74	103.36	0.5	0.5	5.1	105.53	96.49	113.67	NR	66.63	Slightly-different
	Dexketoprofen 1	104.18–109.56	90.46–108.51	84.76–101.47	94.97–112.49	(0.33–4.00)	(0.33–1.00)	0–9.9	87.78–126.87	71.24–130.68	84.39–153.12	NR	55.18–80.46	
	(n = 29)	5.62	20.53	20.36	19.12				43.04	76.53	74.84	NR	44.07	
4		103.01	112.57	109.29	99.38	1.17	1	−7.5	86.97	119.25	119.63	113.68	NR	
	Etoricoxib 2	99.13–107.03	104.27–121.54	100.69–118.61	94.04–105.03	(0.50–3.50)	(0.50–5.00)	−24.9–9.9	69.2–109.29	89.36–159.14	101.49–141	76.24–169.51	NR	
	(n = 34)	9.33	18.78	20.15	13.53				60.27	79.94	41.71	125.13	NR	
11		101.79	102.43	100.63	96.81	1.75	1.625	−3.75	94.74	104.19	109.13	106.41	81.04	
	Ibuprofen 7	98.11–105.60	94.98–110.46	93.81–107.95	89.77–104.39	(0.50–3.50)	(0.75–5.00)	−37.5–22.5	73.5–122.12	65.58–165.51	80.98–147.04	75.50–149.99	58.63–112.03	
	(n = 24)	7.44	15.31	14.26	15.34				54.89	118.29	66.19	78.43	72.95	
14		99.02	89.19	90.08	107.92	1.75	2.125	1.2	105.69	95.31	100.93	104.44	82.28	
	Tadalafil 1	93.69–104.65	82.06–96.95	84.04–96.54	97.7–119.22	(0.67–6.00)	(0.67–6.00)	−24.9–37.5	81.06–137.79	67.92–133.76	79.94–127.44	78.97–138.11	64.77–104.52	
	(n = 34)	13.49	20.47	17.01	24.62				72	98.85	61.72	76.55	63.43	
15		105.92	98.02	92.54	102.81	1.75	2.5	7.5	113.84	85.11	115.49	125.08	100.52	
	Tadalafil 2	101.98–110.01	91.15–105.40	87.34–98.05	98.41–107.42	(0.67–6.00)	(0.67–6.00)	−22.5–32.4	91.06–142.32	64.06–113.09	92.16–144.73	93.61–167.12	84.95–118.96	
	(n = 33)	9.06	17.45	13.93	10.54				57.62	76.88	58.34	78.28	41.85	
17		100.2	104.04	103.83	97.69	3	2.75	−16.2	94.72	108.05	94.86	100.56	70.79	
	Tadalafil 4	95.83–104.78	98.78–109.58	97.96–110.05	93.29–102.29	(1.00–12.00)	(1.67–8.00)	−54.9–17.4	80.64–111.25	86.65–134.74	82.08–109.63	79.98–126.42	51.55–97.20	
	(n = 35)	11.07	12.87	14.47	11.42				41.44	58.95	36.98	61.4	92.07	
3		100.49	118.2	117.62	99.37	1	0.67	−19.8	73.47	148.24	148.58	166.6	144.08	Different
	Etoricoxib 1	97.55–103.52	111.26–125.57	111.48–124.1	94.9–104.06	(0.33–6.00)	(0.33–3.00)	−30.0–−9.9	63.23–85.37	122.39–179.55	126.59–174.4	136.58–203.22	126.56–164.03	
	(n = 48)	8.68	17.78	15.75	13.53				46.02	60.63	49.45	63.22	39.24	
5		103.51	117.32	113.34	95.94	1.25	0.5	−45.0	34.89	465.15	129.91	166	157.16	
	Ibuprofen 1	98.63–108.64	111.33–123.63	106.52–120.58	92.54–99.47	(0.50–3.50)	(0.25–0.75)	−60.0–−37.5	29.8–40.85	369.38–585.76	112.47–150.07	140.77–195.76	147.31–167.66	
	(n = 36)	12.18	13.21	15.66	9.08				41.21	63.1	37.43	43.21	16.34	
7		100.77	76.24	75.66	109.03	0.375	0.5	15.0	152.12	53.65	71.14	79.52	74.42	
	Ibuprofen 3	96.36–105.38	70.46–82.50	71.15–80.45	105.16–113.04	(0.25–2.00)	(0.25–3.50)	0–32.4	116.19–199.16	35.96–80.05	63.51–79.68	70.35–89.87	65.60–84.42	
	(n = 24)	9.05	16.01	12.45	7.31				58.76	96.1	23.23	25.07	25.85	
8		101.57	72.82	71.69	132.75	1.165	2.5	90.0	239.78	20.75	81.64	29.52	25.68	
	Ibuprofen 4	97.23–106.10	67.06–79.07	65.71–78.21	122.83–143.46	(0.33–6.00)	(1.33–6.00)	60.0–120.0	186.78–307.82	13.05–32.99	59.93–111.24	19.52–44.65	17.02–38.76	
	(n = 24)	8.82	16.73	17.72	15.79				53.88	118.68	69.14	100.39	99.63	
9		103.43	98.88	95.60	96.41	2	1.67	−20.1	90	122.52	122.32	142.42	107.36	
	Ibuprofen 5	99.54–107.47	92.83–105.32	89.5–102.11	90.38–102.85	(0.67–4.00)	(0.33–6.00)	−49.8–9.9	66.97–120.95	71.16–210.97	88.81–168.47	107.67–188.39	88.49–130.26	
	(n = 24)	7.74	12.78	13.38	13.11				65.48	153	72.1	61.24	40.53	
10		98.81	89.47	90.55	105.83	2	2.25	30.0	131.39	63.71	73.15	83.51	54.88	
	Ibuprofen 6	95.15–102.61	83.68–95.67	84.78–96.72	98.85–113.29	(0.50–4.00)	(1.00–6.00)	0–60.0	106.5–162.09	43.39–93.55	49.71–107.66	57.34–121.62	38.26–78.72	
	(n = 24)	7.62	13.57	13.38	13.84				44.43	90.96	91.68	88.16	83.55	
16		93.51	85.89	91.85	102.39	3	3	0	105.12	94.79	90.82	94.35	88.09	
	Tadalafil 3	87.98–99.40	79.22–93.11	86.46–97.57	97.58–107.44	(0.67–6.00)	(0.67–6.00)	−20.6–41.2	89.43–123.55	76.02–118.21	75.33–109.49	79.23–112.37	78.47–98.88	
	(n = 30)	13.96	18.54	13.84	10.99				38.11	53.68	44.64	41.42	26.78	
18		94.6	92.08	97.34	103.97	3.5	3.5	7.5	104.85	94.45	89.05	86.26	67.14	
	Tadalafil 5	89.67–99.80	84.71–100.10	90.76–104.4	96.84–111.62	(1.50–8.00)	(1.50–8.00)	−97.5–90.0	73.14–150.31	57.67–154.68	74.3–106.73	68.62–108.42	48.96–92.06	
	(n = 18)	9.21	14.42	12.11	12.29				68.62	103.05	32.01	40.86	58.5	

NR—not reported due to the existence of an individual value of zero, which does not allow the calculation. n—number of subjects.

## Data Availability

Data are available on request due to restrictions (e.g., privacy, legal or ethical reasons).

## Supplementary Materials

The following supporting information can be downloaded at https://www.mdpi.com/article/10.3390/pharmaceutics18080988/s1, Table S1: Point estimate of the test/reference ratio, 90% confidence interval and intra-subject variability of the partial AUC truncated at different sampling times and at Tmax of the reference product in each subject. Empty cells for times where samples were not taken in the corresponding bioequivalence study or far from reference product Tmax; Section S1: Sampling times of the bioequivalence studies.

## Abbreviations

The following abbreviations are used in this manuscript:

ANDA	Abbreviated New Drug Application
AUC	Area under the plasma concentration–time curve
AUC0-t	Area under the curve from time zero to the last quantifiable concentration
AUC0-inf	Area under the curve from time zero extrapolated to infinity
AUMC	Area under the first moment curve
BE	Bioequivalence
Cmax	Maximum plasma concentration
IR	Immediate release
ka	Absorption rate constant
ke	Elimination rate constant
MAT	Mean absorption time
MRT	Mean residence time
pAUC	Partial area under the curve
pAUCtmax	Partial AUC truncated at the population median tmax of the reference product
pAUCRef tmax	Partial AUC truncated at the tmax of the reference product within each individual subject
PSBEG	Product-specific bioequivalence guidance
tlag	Lag time before measurable absorption/concentration
tmax	Time to maximum plasma concentration
