# Supplementary material for: Comparison of Metrics for Investigating Bioequivalence in Onset of Action in Incurred Bioequivalence Studies
Source: Pharmaceutics. 2026 Aug 10;18(8):988. doi: 10.3390/pharmaceutics18080988 (PMC13516050; doi:10.3390/pharmaceutics18080988)
Supplement: Supplementary file 1 [file pharmaceutics-18-00988-s001.zip › pharmaceutics-4465187-supplementary.pdf]

Table S1. Point estimate of the test/reference ratio, 90% confidence interval and intra-subject variability of the partial AUC truncated at different sampling times and at Tmax of the reference product in each subject. Empty cells for times where samples were not taken in the corresponding bioequivalence study or far from reference product Tmax.

Time scale for AUC	Drug	Reference Tmax Median (range)	Test Tmax Median (range)	A: $AUC_{0.25\ h}$	A: $AUC_{0.33\ h}$	A: $AUC_{0.50\ h}$	A: $AUC_{0.67\ h}$	A: $AUC_{0.75\ h}$	A: $AUC_{0.83\ h}$	A: $AUC_{1.00\ h}$	A: $AUC_{1.25\ h}$	A: $AUC_{1.33\ h}$	A: $AUC_{1.50\ h}$	A: $AUC_{1.67\ h}$	A: $AUC_{1.75\ h}$	A: $AUC_{2.00\ h}$	$AUC_{Ref-Tmax}$
				B: $AUC_{1.00\ h}$	B: $AUC_{0-1.25\ h}$	B: $AUC_{0-1.33\ h}$	B: $AUC_{0-1.5\ h}$	B: $AUC_{0-1.67\ h}$	B: $AUC_{0-1.75\ h}$	B: $AUC_{0-2\ h}$	B: $AUC_{0-2.25\ h}$	B: $AUC_{0-2.5\ h}$	B: $AUC_{0-2.75\ h}$	B: $AUC_{0-3\ h}$	B: $AUC_{0-3.33\ h}$	B: $AUC_{0-3.5\ h}$	
A:	Dexketoprofen 2	0.67<br>(0.33-1.75)	0.67<br>(0.33-3.00)			78.57<br>61.66-100.12<br>65.67	**85.01**<br>**69.86-103.44**<br>**51.48**		91.53<br>78.46-106.77<br>39.48	94.82<br>83.50-107.67<br>32.20							**81.32**<br>**67.92-97.35**<br>**46.75**
A:	Ibuprofen 2	0.75<br>(0.33-2.50)	1.00<br>(0.33-2.00)		169.13<br>118.18-242.04<br>82.88	149.22<br>113.41-196.34<br>59.88		**129.48**<br>**101.79-164.70**<br>**51.54**		120.31<br>95.60-151.41<br>48.99	117.72<br>94.01-147.40<br>47.80						**107.31**<br>**91.60-125.71**<br>**32.76**
A:	Metamizole	1.00<br>(0.67-2.67)	1.125<br>(0.67-2.33)				84.73<br>71.39-100.57<br>35.62			**89.39**<br>**79.50-100.50**<br>**23.97**	92.34<br>84.47-100.94<br>18.11		94.35<br>88.04-101.12<br>14.05		95.36<br>90.00-101.04<br>11.71		**86.22**<br>**76.86-96.73**<br>**23.51**
A:	Paracetamol	0.50<br>(0.17-2.00)	0.50<br>(0.33-1.25)		114.10<br>83.73-155.50<br>69.06	**111.19**<br>**88.61-139.51**<br>**48.29**	105.86<br>90.39-123.98<br>32.70			103.57<br>91.64-117.06<br>25.08							**85.23**<br>**63.90-113.68**<br>**63.36**
A:	Tramadol	0.75<br>(0.50-1.67)	0.75<br>(0.50-2.00)			105.11<br>81.69-135.24<br>54.33		**100.93**<br>**84.85-120.06**<br>**36.11**		100.75<br>88.17-115.12<br>27.40		102.47<br>92.10-114.00<br>21.77		103.86<br>94.79-113.80<br>18.59			**99.95**<br>**82.48-121.12**<br>**40.26**
A:	Vardenafil	0.75<br>(0.33-2.33)	0.75<br>(0.33-2.33)			133.20<br>93.49-189.78<br>128.26		**119.12**<br>**90.44-156.88**<br>**89.53**		111.74<br>89.48-139.53<br>68.32	106.82<br>89.41-127.61<br>52.76						**90.58**<br>**73.14-112.18**<br>**65.28**
A:	Dexketoprofen 1	0.50<br>(0.33-4.00)	0.50<br>(0.33-1.00)		NR<br>NR<br>NR	**NR**<br>**NR**<br>**NR**	107.86<br>69.14-168.29<br>129.80		112.43<br>77.49-163.14<br>99.85	114.10<br>82.73-157.37<br>82.18							**66.63**<br>**55.18-80.46**<br>**44.07**
A:	Etoricoxib 2	1.17<br>(0.50-3.50)	1.00<br>(0.50-5.00)					122.31<br>73.40-203.81<br>191.37		**113.68**<br>**76.24-169.51**<br>**125.13**		111.55<br>82.73-150.40<br>83.34		111.22<br>87.82-140.86<br>62.47		109.72<br>91.61-131.40<br>46.02	**NR**<br>**NR**<br>**NR**
B:	Ibuprofen 7	1.75<br>(0.50-3.50)	1.625<br>(0.75-5.00)	116.00<br>74.20-181.33<br>111.94	114.49<br>75.74-173.07<br>100.17		109.62<br>75.18-159.83<br>88.55		**106.41**<br>**75.50-149.99**<br>**78.43**	105.18<br>77.62-142.51<br>67.51		104.04<br>81.91-132.13<br>51.18					81.04<br>58.63-112.03<br>72.95
B:	Tadalafil 1	1.75<br>(0.67-6.00)	2.125<br>(0.67-6.00)	113.81<br>79.22-163.51<br>108.20	110.12<br>79.49-152.55<br>93.41		106.87<br>78.98-144.60<br>84.60		**104.44**<br>**78.97-138.11**<br>**76.55**	102.80<br>79.41-133.07<br>69.43	101.95<br>80.30-129.43<br>63.24						82.28<br>64.77-104.52<br>63.43
B:	Tadalafil 2	1.75<br>(0.67-6.00)	2.5<br>(0.67-6.00)	NR<br>NR<br>NR	136.63<br>95.29-195.90<br>104.60		128.03<br>93.31-175.66<br>87.61		**125.08**<br>**93.61-167.12**<br>**78.28**	123.27<br>94.65-160.54<br>69.83	121.35<br>95.59-154.05<br>61.86						100.52<br>84.95-118.96<br>41.85
B:	Tadalafil 4	3.00<br>(1.00-12.00)	2.75<br>(1.67-8.00)	77.95<br>41.57-146.17<br>318.90		84.79<br>49.19-146.17<br>226.16		91.31<br>56.51-147.55<br>175.49		96.50<br>65.54-142.08<br>122.20	98.19<br>70.45-136.87<br>98.01	98.77<br>73.82-132.15<br>82.35	99.87<br>77.17-129.24<br>70.76	**100.56**<br>**79.98-126.42**<br>**61.40**	100.94<br>82.77-123.10<br>52.14		70.79<br>51.55-97.20<br>92.07
A:	Etoricoxib 1	1.00<br>(0.33-6.00)	0.67<br>(0.33-3.00)		234.07<br>176.48-310.46<br>98.62		190.00<br>150.33-240.14<br>77.17			**166.60**<br>**136.58-203.22**<br>**63.22**		149.95<br>127.15-176.84<br>51.07					**144.08**<br>**126.56-164.03**<br>**39.24**
A:	Ibuprofen 1	1.25<br>(0.50-3.50)	0.50<br>(0.25-0.75)					263.55<br>197.46-351.75<br>83.06		197.30<br>159.87-243.49<br>56.68	**166.00**<br>**140.77-195.76**<br>**43.21**		148.39<br>129.19-170.46<br>35.86		132.27<br>121.55-155.03<br>31.25		**157.16**<br>**147.31-167.66**<br>**16.34**
A:	Ibuprofen 3	0.375<br>(0.25-2.00)	0.5<br>(0.25-3.50)	79.44<br>69.68-90.58<br>26.93		**79.52**<br>**70.35-89.87**<br>**25.07**		79.94<br>70.62-90.49<br>25.41		81.09<br>72.03-91.28<br>24.22							**74.42**<br>**65.60-84.42**<br>**25.85**
A:	Ibuprofen 4	1.165<br>(0.33-6.00)	2.50<br>(1.33-6.00)				24.64<br>14.86-40.85<br>135.23			25.55<br>16.30-40.05<br>112.97		**29.52**<br>**19.52-44.65**<br>**100.39**		35.34<br>24.56-50.86<br>84.56		40.76<br>29.56-56.21<br>72.28	**25.68**<br>**17.02-38.76**<br>**99.63**
B:	Ibuprofen 5	2.00<br>(0.67-4.00	1.67<br>(0.33-6.00)	245.54<br>137.79-437.55<br>170.00		175.41<br>119.44-257.61<br>90.78		158.95<br>113.15-223.29<br>77.48		**142.42**<br>**107.67-188.39**<br>**61.24**		125.10<br>101.59-154.04<br>43.91		115.63<br>98.57-135.65<br>33.06			107.36<br>88.49-130.26<br>40.53

Time scale for AUC	Drug	Reference Tmax Median (range)	Test Tmax Median (range)	A: $AUC_{0.25\ h}$	A: $AUC_{0.33\ h}$	A: $AUC_{0.50\ h}$	A: $AUC_{0.67\ h}$	A: $AUC_{0.75\ h}$	A: $AUC_{0.83\ h}$	A: $AUC_{1.00\ h}$	A: $AUC_{1.25\ h}$	A: $AUC_{1.33\ h}$	A: $AUC_{1.50\ h}$	A: $AUC_{1.67\ h}$	A: $AUC_{1.75\ h}$	A: $AUC_{2.00\ h}$	$AUC_{Ref-Tmax}$
				B: $AUC_{1.00\ h}$	B: $AUC_{0-1.25\ h}$	B: $AUC_{0-1.33\ h}$	B: $AUC_{0-1.5\ h}$	B: $AUC_{0-1.67\ h}$	B: $AUC_{0-1.75\ h}$	B: $AUC_{0-2\ h}$	B: $AUC_{0-2.25\ h}$	B: $AUC_{0-2.5\ h}$	B: $AUC_{0-2.75\ h}$	B: $AUC_{0-3\ h}$	B: $AUC_{0-3.33\ h}$	B: $AUC_{0-3.5\ h}$	
**B:**	Ibuprofen 6	2.00<br>(0.50-4.00)	2.25<br>(1.00-6.00)	90.22<br>56.90-143.04<br>117.22	82.90<br>51.99-132.19<br>119.38		81.88<br>52.29-128.21<br>112.55			**83.51**<br>**57.34-121.62**<br>**88.16**		86.21<br>63.54-116.97<br>67.86		89.06<br>71.19- 111.43<br>47.60		90.98<br>77.30-107.09<br>33.80	54.88<br>38.26-78.72<br>83.55
**B:**	Tadalafil 3	3<br>(0.67-6.00)	3<br>(0.67-6.00)		98.77<br>69.72-139.94<br>93.60		98.68<br>72.29-134.71<br>80.75		98.77<br>74.87-130.31<br>69.91	97.76<br>76.24-125.35<br>61.45	96.26<br>76.82-120.63<br>54.95	95.23<br>77.48-117.04<br>49.66	94.74<br>78.43-114.44<br>45.08	**94.35**<br>**79.23-112.37**<br>**41.42**	92.96<br>79.86-108.21<br>35.64		88.09<br>78.47-98.88<br>26.78
**B:**	Tadalafil 5	3.50<br>(8.00-1.50)	3.50<br>(8.00-1.50)		94.42<br>42.08-207.37<br>235.31		89.87<br>49.61-162.79<br>135.49		87.04<br>52.75-143.63<br>104.75	85.52<br>55.58-131.61<br>85.49	83.95<br>57.97-121.57<br>70.65	83.35<br>60.12-115.55<br>60.86	83.90<br>62.58-112.48<br>53.75	84.74<br>64.92-110.60<br>48.28		**86.26**<br>**68.62-108.42**<br>**40.86**	67.14<br>48.96-92.06<br>58.50

Section S1. Sampling times of the bioequivalence studies.

Median reference product Tmax in bold. Median test product Tmax in italics. If the median occurs in between two samples both are highlighted.

1- Dexketoprofen 1

Baseline, 0.17, 0.33, ***0.50***, 0.67, 0.83, 1.00, 1.25, 1.50, 1.75, 2.00, 2.50, 3.00, 4.00, 5.00, 6.00, 8.00, 10.00 and 12.00 hours post-dose

2- Dexketoprofen 2

Baseline, 0.17, 0.33, 0.50, ***0.67***, 0.83, 1.00, 1.25, 1.50, 1.75, 2.00, 2.50, 3.00, 4.00, 6.00, 8.00, 10 and 12 hours post-dose

3- Etoricoxib 1

Baseline, 0.33, *0.67*, **1.00** 1.33, 1.67, 2.00, 2.33, 2.67, 3.00, 3.50, 4.00, 6.00, 8.00, 10.00, 12.00, 24.00, 48.00 and 72.00 hours post-dose

4- Etoricoxib 2

Baseline, 0.25, *0.50*, 0.75, ***1.00*, 1.33,** 1.67, 2.00, 2.33, 2.67, 3.00, 3.50, 4.00, 5.00, 6.00, 8.00, 10.00, 12.00, 24.00, 48.00 and 72.00 hours post-dose

5- Ibuprofen 1

Baseline, 0.25, 0.50, 0.75, 1.00, **1.25**, 1.50, 1.75, 2.00, 2.50, 3.00, 3.50, 4.00, 5.00, 6.00, 8.00, 10.00 and 12.00 hours post-dose.

6- Ibuprofen 2

Baseline, 0,08, 0.17, 0,33, 0.50, **0.75**, *1.00*, 1.25, 1.50, 2.00, 2.50, 3.00, 3.50, 4.00, 6.00, 8.00, 10.00 and 12.00 hours post-dose

7-Ibuprofen 3

Baseline, **0.25, *0.50***, 0.75, 1.00, 1.33, 1.67, 2.00, 2.50, 3.00, 3.50, 4.00, 5.00, 6.00, 8.00, 10.00, 12.00, 14.00 and 24.00 hours post-dose

8- Ibuprofen 4

Baseline, 0.33, 0.67, **1.00, 1.33**, 1.67, 2.00, *2.50*, 3.00, 4.00, 5.00, 6.00, 8.00, 10.00, 12.00, 15.00 and 22.00 hours post-dose

9- Ibuprofen 5

Baseline, 0.33, 0.67, 1.00, 1.33, *1.67*, **2.00**, 2.50, 3.00, 4.00, 5.00, 6.00, 8.00, 10.00 and 12.00 hours post-dose

10- Ibuprofen 6

Baseline, 0.25, 0.50, 0.75, 1.00, 1.25, 1.50, ***2.00****, 2.50*, 3.00, 3.50, 4.00, 5.00, 6.00, 8.00, 10.00 and 12.00 hours post-dose

11- Ibuprofen 7

Baseline, 0.25, 0.50, 0.75, 1.00, 1.25, *1.50,* ***1.75***, 2.00, 2.50, 3.00, 3.50, 4.00, 5.00, 6.00, 8.00, 10.00 and 12.00 hours post-dose

12- Metamizole

Baseline, 0.33, 0.67, ***1.00****, 1.25*, 1.50, 1.75, 2.00, 2.33, 2.67, 3.00, 3.50, 4.00, 6.00, 8.00, 10.00, 12.00 and 22.00 hours post-dose

13- Paracetamol

Baseline, 0.08, 0.17, 0.33, ***0.50***, 0.75, 1.00, 1.25, 1.50, 2.00, 2.50, 3.00, 3.50, 4.00, 6.00, 8.00, 10.00 and 12.00 hours post-dose

14- Tadalafil 1

Baseline, 0.33, 0.67, 1.00, 1.25, 1.50, **1.75**, *2.00, 2.25*, 2.50, 2.75, 3.00, 3.50, 4.00, 4.50, 5.00, 6.00, 8.00, 12.00, 24.00, 48.00 and 72.00 hours post-dose

15- Tadalafil 2

Baseline, 0.33, 0.67, 1.00, 1.25, 1.50, **1.75**, 2.00, 2.25, *2.50*, 2.75, 3.00, 3.50, 4.00, 5.00, 6.00, 8.00, 12.00, 24.00, 48.00 and 72.00 hours post-dose

16- Tadalafil 5

Baseline, 0.33, 0.67, 1.00, 1.25, 1.50, 1.75, 2.00, 2.25, 2.50, 2.75, ***3.00***, 3.50, 4.00, 5.00, 6.00, 8.00, 12.00, 24.00, 48.00 and 72.00 hours post-dose

17- Tadalafil 4

Baseline, 0.50, 1.00, 1.33, 1.67, 2.00, 2.25, 2.50, *2.75,* **3.00***,* 3.33, 3.67, 4.00, 4.00,5, 5.00, 6.00, 8.00, 12.00, 24.00, 48.00 and 72.00 hours post-dose

18- Tadalafil 5

Baseline, 0.33, 0.67, 1.00, 1.25, 1.50, 1.75, 2.00, 2.25, 2.50, 2.75, 3.00, ***3.50***, 4.00, 5.00, 6.00, 8.00, 12.00, 24.00, 48.00 and 72.00 hours post-dose

19-Tramadol

Baseline, 0.25, 0.50, ***0.75***, 1.00, 1.33, 1.67, 2.00, 2.50, 3.00, 3.50, 4.00, 5.00, 6.00, 8.00, 10.00, 12.00, 14.00 and 24.00 hours post-dose

20- Vardenafil

Baseline, 0.08, 0.17, 0.33, 0.50, ***0.75***, 1.00, 1.25, 1.50, 1.75, 2.00, 2.33, 2.67, 3.00, 3.50, 4.00, 6.00, 8.00, 10.00, 12.00, 14.00, 24.00 and 32.00 hours post-dose
